# Improvement in Rubber Plates

**Published:** 1861-12

**Authors:** J. A. M’Clelland


### IMPROVEMENT IN RUBBER PLATES.

BY J. A. M'CLELLAND, D. D. S.

THEORIES are of no value until tested. In view of this, I suggest a plan, to those who wish to experiment, for putting up rubber plates, which it is thought will prevent their cracking and impart greater strength, while the plates may be made even thinner than they are now made.

The idea originated with me in October, 1860, and was suggested from the frequent applications to repair cracked rubber plates, but having no experimental knowledge in this style of work, the plan has not been tried. It is as follows: Make a skeleton plate of woven wire cloth. (Platina wire would probably be the best, though the experiment might be made with common wire web.) The wire should be of suitable size, and not too closely woven. Fit it as well to the plaster model as possible, place the rubber over it, and proceed with the work in the usual manner. The rubber would be forced through the wire plate sufficiently to make a perfect adaptation to the cast, thus connecting the rubber on the two sides of the skeleton plate.

